# Effects of a physical activity and endometriosis-based education program delivered by videoconference on endometriosis symptoms: the CRESCENDO program (inCRease physical Exercise and Sport to Combat ENDOmetriosis) protocol study

**DOI:** 10.1186/s13063-023-07792-1

**Published:** 2023-11-27

**Authors:** Géraldine Escriva-Boulley, Charles-André Philip, Sophie Warembourg, Lionel Lenotre, Patrice Flore, Patrice Faure, Thierry Michy, Vincent Letouzey, Carole Arnold, Claire Piluso, Loic Chalmel, Ramzi Kacem, Georges Fabrice Blum, Renaud Detayrac, Candice Trocmé, Isabelle Brigaud, Ulysse Herbach, Patricia Branche, Emilie Faller, Aïna Chalabaev

**Affiliations:** 1 Université de Haute-Alsace, Université de Strasbourg, Université de Lorraine, LISEC UR 2310, F-68100 Mulhouse, France; 2 Clinique gynécologique et obstétricale, Hôpital de la Croix-Rousse, groupe hospitalier Nord, CHU de Lyon–HCL, 103, grande rue de la Croix-Rousse, 69317 Lyon cedex, France; 3 Faculté des Sciences et Techniques, Université de Haute-Alsace, 18 Rue des Frères Lumière, 68200 Mulhouse, France; 4 PASTA - Processus aléatoires spatio-temporels et leurs applications, Inria Nancy - Grand Est, Villers-lès-, Nancy, France; 5 Univ. Grenoble Alpes, Inserm, CHU Grenoble Alpes, HP2, 38000 Grenoble, France; 6 Grenoble University Hospital, Avenue Maquis du Grésivaudan, 38700 La Tronche, France; 7 Department of Gynecology, Grenoble University Hospital, Avenue Maquis du Grésivaudan, 38700 La Tronche, France; 8 Service de Gynécologie Obstétrique, Nîmes, France; 9 Université de Lorraine, Inserm, UMRS 1256, NGERE – Nutrition, Genetics, and Environmental Risk Exposure, F-54000 Nancy, France; 10 Université de Haute-Alsace, Université de Strasbourg, SAGE, F-68100 Mulhouse, France; 11 Service gynécologie GHRMSA, Hôpital Emile Muller, Mulhouse, France; 12 Cabinet Médical, Clinique du Diaconat-Fonderie et Université de Haute-Alsace, Mulhouse, France; 13 Université de Haute-Alsace, CNRS, IS2M UMR 7361, Mulhouse, France; 14 Université de Lorraine, CNRS, Inria, IECL, F-54000 Nancy, France; 15 Service d’Anesthésie Réanimation Chirurgicale, Hospices Civils de Lyon, Groupement Hospitalier Nord Hôpital de la Croix-Rousse, 103 Grande Rue de la Croix-Rousse, F-69317 Lyon, France; 16 Department of Gynecologic Surgery, Hôpitaux Universitaires de Strasbourg, 67200 Strasbourg, France; 17 Univ. Grenoble Alpes, SENS, 38000 Grenoble, France

**Keywords:** Physical activity, Endometriosis, Videoconference, RCT, Pelvic pain, Motivational theories, Psychosocial variables

## Abstract

**Background:**

Endometriosis is a chronic disease characterized by growth of endometrial tissue outside the uterine cavity which could affect 200 million women (The term “woman” is used for convenience. Individuals gendered as man or as nonbinary can also suffer from this disease) worldwide. One of the most common symptoms of endometriosis is pelvic chronic pain associated with fatigue. This pain can cause psychological distress and interpersonal difficulties. As for several chronic diseases, adapted physical activity could help to manage the physical and psychological symptoms. The present study will investigate the effects of a videoconference-based adapted physical activity combined with endometriosis-based education program on quality of life, pain, fatigue, and other psychological symptoms and on physical activity.

**Methods:**

This multicentric randomized-controlled trial will propose to 200 patients with endometriosis to be part of a trial which includes a 6-month program with 45 min to more than 120 min a week of adapted physical activity and/or 12 sessions of endometriosis-based education program. Effects of the program will be compared to a control group in which patients will be placed on a waiting list. All participants will be followed up 3 and 6 months after the intervention. None of the participants will be blind to the allocated trial arm. The primary outcome measure will be quality of life. Secondary outcomes will include endometriosis-related perceived pain, fatigue, physical activity, and also self-image, stereotypes, motivational variables, perceived support, kinesiophobia, basic psychological need related to physical activity, and physical activity barriers. General linear models and multilevel models will be performed. Predictor, moderator, and mediator variables will be investigated.

**Discussion:**

This study is one of the first trials to test the effects of a combined adapted physical activity and education program for improving endometriosis symptoms and physical activity. The results will help to improve care for patients with endometriosis.

**Trial registration:**

ClinicalTrials.gov, NCT05831735. Date of registration: April 25, 2023

**Supplementary Information:**

The online version contains supplementary material available at 10.1186/s13063-023-07792-1.

## Administrative information

Note: the numbers in curly brackets in this protocol refer to SPIRIT checklist item numbers. The order of the items has been modified to group similar items (see http://www.equator-network.org/reporting-guidelines/spirit-2013-statement-defining-standard-protocol-itemsfor-clinical-trials/).

**Table:** 

Title {1}	Effects of a physical activity and endometriosis-based education program delivered by videoconference on endometriosis symptoms: the CRESCENDO program (inCRease physical Exercise and Sport to Combat ENDOmetriosis) protocol study
Trial registration {2a and 2b}.	ClinicalTrials.gov, Identifier NCT05831735. Date of registration: 04/25/2023
Protocol version {3}	First version (dated 25/04/2023)
Funding {4}	National Research Agency [Agence Nationale de la Recherche], association EndoFrance. This trial is non-commercial. These are external funding.
Author details {5a}	^1^ Université de Haute-Alsace, Université de Strasbourg, Université de Lorraine, LISEC UR 2310, F-68100 Mulhouse, France ^2^ Clinique gynécologique et obstétricale, hôpital de la Croix-Rousse, groupe hospitalier Nord, CHU de Lyon–HCL, 103, grande rue de la Croix-Rousse, 69317 Lyon cedex, France ^3^ Faculté des Sciences et Techniques, Université de Haute-Alsace, 18 Rue des Frères Lumière, 68200 Mulhouse. ^4^ PASTA - Processus aléatoires spatio-temporels et leurs applications, Inria Nancy - Grand Est ^5^ Univ. Grenoble Alpes, Inserm, CHU Grenoble Alpes, HP2, 38000 Grenoble, France ^6^ Grenoble University hospital, Avenue Maquis du Grésivaudan, 38700 La Tronche, France ^7^ Department of gynecology, Grenoble University hospital, Avenue Maquis du Grésivaudan, 38700 La Tronche, France ^8^ Service de Gynécologie Obstétrique, Nîmes, France ^9^ Université de Lorraine, Inserm, UMRS 1256, NGERE – Nutrition, Genetics, and Environmental Risk Exposure, F-54000 Nancy, France ^10^ Université de Haute-Alsace, Université de Strasbourg, SAGE, F-68100 Mulhouse, France ^11^ Service gynécologie GHRMSA, Hôpital Emile Muller, Mulhouse, France ^12^ Cabinet Médical, Clinique du Diaconat-Fonderie et Université de Haute-Alsace, Mulhouse, France ^13^ Université de Haute-Alsace, CNRS, IS2M UMR 7361 ^14^ Université de Lorraine, CNRS, Inria, IECL, F-54000 Nancy, France ^15^ Service d’Anesthésie Réanimation Chirurgicale, Hospices Civils de Lyon, Groupement Hospitalier Nord Hôpital de la Croix-Rousse, 103 Grande Rue de la Croix-Rousse, Lyon F-69317, France ^16^ Department of Gynecologic Surgery, Hôpitaux Universitaires de Strasbourg, 67200 Strasbourg, France ^17^ Univ. Grenoble Alpes, SENS, 38000 Grenoble, France
Name and contact information for the trial sponsor {5b}	National Research Agency tristan.lescure@agencerecherche.fr EndoFrance contact@endofrance.org
Role of sponsor {5c}	The funders have no role in study neither in design, analysis, interpretation, or publication of the study protocol and trial results.

## Introduction

### Background and rationale {6a}

Endometriosis is a chronic disease characterized by growth of endometrial tissue outside the uterine cavity which affects approximately 10% of women of reproductive age worldwide [1]. One of the most reported symptoms of endometriosis is pelvic chronic pain, associated with fatigue. Infertility could also be one of the symptoms. These symptoms can cause psychological distress and interpersonal issues that are as difficult to live with as the pain itself. Compared to women who do not suffer from endometriosis, women with this disease develop a lower sense of femininity, altered body image, higher levels of stress, depression, and anxiety [2, 3]. These factors affect their QoL [4] and can have negative impacts on the response to treatment [5]. Interpersonal relationships are also impacted such as romantic [6, 7], professional [8], and social relationships [9], because the symptoms make it difficult to move and carry out daily activities [10].

This disease generates significant health care costs (e.g., 9.5 billion in France, 7.4 billion in Australia) because there is no curative therapy for it, and its management involves long-term pain-related pharmaceutical treatment and/or iterative surgery, which remain palliative care [11]. However, research showed that these treatments are only relatively effective in reducing pain [12, 13] and that this disease or its symptoms have high recurrence rates (e.g., 2.7% to 6.3% of rehospitalization after surgery at least one at 1 year [14]). These results invited to go further and to include complementary therapies as an adjunction in the pain management to increase health care efficacy [15].

Recent articles highlighted the beneficial effects of regular physical activity (PA) and adapted physical activity (APA) on chronic diseases [16, 17]. Research suggested that (A)PA may have a beneficial effect on the physical symptoms and the psychological and social consequences of these diseases [18, 19], thus maybe also in endometriosis context. However, studies questioning the link between PA and endometriosis (and its symptoms) are rare, mostly cross-sectional, and their results are inconsistent [20–22]. The scarcity of studies can be explained by the painful symptoms caused by the disease, leading women not to practice much PA spontaneously and autonomously or even avoid practicing. In addition to this difficulty, widely recognized barriers to PA (i.e., lack of time, lack of adequate and nearby infrastructure, and low self-confidence and motivation for PA) can also contribute to the low level of PA [16]. Otherwise, due to their cross-sectional design, it was not possible for these studies to establish a causal link between PA and endometriosis-related symptoms. Randomized controlled trials (RCT) are needed to test the effects of (A)PA on the endometriosis symptoms and its consequences.

Prior report highlighted the beneficial effects of regular PA and particularly PA adapted to motor abilities with regard to the disease, called APA, on chronic diseases [16]. What differentiates PA from APA is that in addition to being based on health recommendations, APA is offered by qualified personnel and includes motivational and educational components aimed at promoting a healthy lifestyle. (A)PA appears to reduce pain severity and improve physical functioning [19]—particularly through its anti-inflammatory effects [16]—reduce fatigue [23, 24], and enhance psychological and social health [18].

In endometriosis setting, in vivo experiments on animal models such as mice and rats have tested the effects of PA. Results showed that PA may reduce oxidative stress, reduce the development of the disease and the size of endometriotic lesions, and has an anti-inflammatory effect (e.g., [25–27]). To our knowledge, six other studies (RCT) investigated the effects of PA on endometriosis and endometriosis symptoms on humans. In two clinical trials (NCT03994432 and NCT05091268), researchers investigated the role of PA, specifically. They evaluate whether a dietary recommendation based on the Mediterranean diet’s principles associated with a regular aerobic PA, according to the “7 minutes workout” (12 high-intensity physical exercises, lasting 30 s each and spaced out 10 s of short breaks, to be performed 2–3 times a week), may improve pain symptoms in patients with symptomatic endometriosis, treated with estrogen-progestins or progestins (*N* = 140). The choice of this type of PA is based on one well-known barrier of PA, that is, the lack of time. They also aim to study the effect of pain education and group-based PA versus pain education alone on women with endometriosis-associated pain. Participants (*N* = 78) will all attend a four-hour pain education session, while the intervention group also receives a 60-min weekly group training session led by a physiotherapist and a progressive home exercise program performed daily over a period of 4 months. Exercise will focus on general strength and cardiovascular fitness, stretching, and relaxation (NCT05091268). Results from those studies have not been published yet. Two protocol studies [28, 29] planned (I) to explore the potential benefits of a therapeutic exercise program on the health-related QoL (*N* = 26) and (II) to assess the efficacy of yoga and cognitive–behavioral therapy, above education, on quality of life, biopsychosocial outcomes, and cost-effectiveness (*N* = 228), respectively. Another study [30] tested the effect of a 24-week PA program on patients who benefited from a medical treatment (*N* = 36). The results showed a decrease of the treatment side effects in the PA group (muscle cramps, back pain, headache, fatigue) but no significant difference in psychological symptoms or time to recurrence. Finally, a pilot study (*N* = 22) aimed to investigate the impact of a single session of “supervised” telehealth-delivered exercise compared to “self-managed” virtual reality (VR)-delivered exercise on pelvic pain [31]. The “supervised” telehealth-delivered exercise consisted in a 1-h supervised session that included cardiorespiratory exercise (interval training) and stretching. The “self-managed” virtual reality (VR)-delivered exercise consisted in a 1-h unsupervised session with (i) a 10-min VR pain-distraction experience and (ii) 50 min of exercise using a PA application. These interventions were compared to a control group (non-intervention). Results showed no difference between groups but highlighted a more favorable score for the groups with intervention compared to the control group. The PA proposed in these studies was not APA.

It is worth noticing that only three of these studies used educational and/or psychological sessions to be part or complement of PA sessions in their program. However, it has been proven that PA program accompanied by an educational program is more effective [16, 32, 33]. Furthermore, none of these studies make it possible to assess the specific role and mechanisms of action of each component of the program (e.g., APA, educational sessions), which makes it impossible to identify the effective leverages of intervention. Otherwise, for each of these studies, all or part of participants need to go on site to benefit from the PA program. However, barriers of PA are related to time, proximity, accessibility, and, in chronic diseases, to some symptoms (i.e., pain, fatigue that can reduce the ability to drive or commute to the places in which PA sessions are delivered). In this context, new technologies could be a promising lever. APA delivered via videoconferencing showed lower attrition, a good completion rate, and high satisfaction with the program [34]. What participants enjoyed were the organizational simplicity and the possibility to hide the disease and build a social link [35]. The low number of results from RCT studies which did not propose post-intervention follow-up, the cross-sectional design of most studies about PA and endometriosis, led us to propose the CRESCENDO (inCRease physical Exercise and Sport to Combat ENDOmetriosis) program, an RCT, with 6 months follow-up, to test the effects of combined (A)PA and endometriosis-based education delivered via videoconference on endometriosis symptoms and its consequences.

### Objectives {7}

The trial objectives will be (I) to test the effects of a “APA + endometriosis-based education” in improving QoL, but also in decreasing perceived pain and fatigue and in increasing PA, (II) to investigate potential predictor and mediator/moderator variables of treatment outcomes, and (III) to help in improving medical, physical, and psychological care for patients by proposing a structured program and by using videoconference to reduce barriers to PA.

### Trial design {8}

The study will be a multicentric randomized-controlled superiority trial which will include four arms (i.e., “APA,” “education,” “APA + education,” and “waitlist control group”) and will test the effect of a 6-month program on QoL in patients with endometriosis. The efficacy of the program in intervention groups will be compared to a control group (i.e., waitlist group control) which will benefit from APA and education sessions at the end of the 6 months program. Randomization will be balanced with a 1:1:1:1 ratio for patients’ allocation to the three intervention groups and the waitlist control group. Identifying potential moderators and mediators of treatment outcome will also be a component of this trial.

## Methods: participants, interventions, and outcomes

### Study setting {9}

**Fig. 1:**
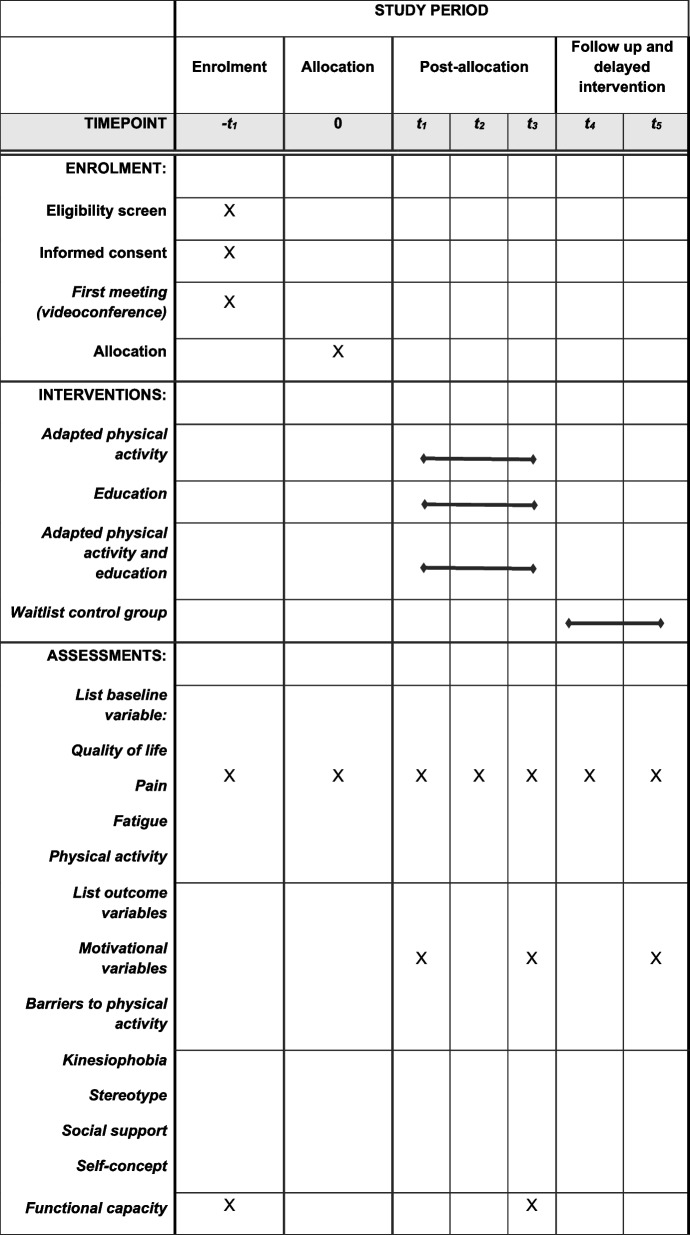
SPIRIT study schedule. *t*_5_ is the last time of measurement and the endline assessment

Patients with endometriosis will be recruited in French speaking countries (e.g., France, Canada, Switzerland, Belgium). Participants will use their own new technology devices (e.g., notebook, PC, tablet, laptop) to access and attend the videoconferencing APA and/or education sessions. The schedule of the study is presented in Fig. 1.

### Eligibility criteria {10}

The inclusion criteria are as follows:

- Endometriosis diagnosed (by physical exam, MRI)
- Age > 18 and sexually active
- Moderate to significant functional difficulties, pain, and fatigue (between 4 and 10/10 points on visual analogue scale)
- Sufficient French language skills
- Free access to the Internet with a stable Internet connection on a device which allow to see movement and be seen (webcam)

The exclusion criteria are as follows:

- Disabling disease other than endometriosis with the same major symptoms (cancer, arthritis, ...)
- BMI ≥ 40
- No major difficulties related to the disease
- Participation to another program which propose education and/or PA program at the same time that the CRESCENDO program
- Surgery or medically assisted procreation scheduled within 9 months

### Who will take informed consent? {26a}

Informed consent will be managed by the principal investigator (PI). Patients will be informed by their endometriosis specialists in one of the French health centers partners to this program (i.e., La Croix Rousse Hospital Lyon, Strasbourg Hospital, Grenoble Hospital, Nimes Hospital, Mulhouse Hospital) of the existence of the program, and the latter will give them a flyer of the program with the PI’s email. Once the email is received, the PI will send the consent form containing written information about study (e.g., aim, duration, data collections during and after the program, randomization process) and will invite the potential participants to discuss the program via a one-to-one scheduled videoconference (will also allow to ensure the technology and Internet access). This first videoconference contact will last 30 min. This meeting will allow to extensively present the study and answer potential participant’s questions. Then, participants will be asked to send written informed consent when they want (within a few hours, days, or weeks) to ensure that consent is given without any pressure and to leave time to read again the written information.

### Additional consent provisions for collection and use of participant data and biological specimens {26b}

This trial does not involve collecting biological specimens for storage.

## Interventions

### Explanation for the choice of comparators {6b}

The comparator will be the waitlist control group. As much as all participants, those in the waitlist control group will continue with their usual treatment and activities during their participation to the study (e.g., hormonal treatment, antalgic treatment, PA, physiotherapy). The allocation in a group being done randomly, and because each endometriosis case is unique in terms of symptoms and treatment, using a waitlist control seemed relevant to compare the effects of the interventions on participants with similar characteristics. For ethical reasons, the waitlist control group will benefit from the intervention after the and on the main intervention (i.e., 6 months after they start the program).

### Intervention description {11a}

All participants in the study will receive a personalized PA assessment (physical tests) after inclusion, before the start of the program. This assessment will be conducted by an APA teacher. A booklet on PA recommendations, in particular the health benefits of PA and the latest studies on PA and endometriosis, will be given to all participants. This booklet will also include a link to a video of movements to perform. Participants will receive a second PA assessment at 6 months (end of the intervention).

### Description of the “APA” arm

In addition to PA recommendations, participants randomized to this arm will receive PA sessions via videoconference for 6 months. The sessions will be based on the personalized PA assessment and will last between 45 min (at the beginning of the program) and 1 h, 1 to 3 times a week, supervised or on their own. Participants will benefit from this program free of charge (Table 1).

**Table 1:** Duration and type of PA proposed

Physical activity	Month #1	Month #2	Month #3	Month #4	Month #5	Month #6
Pilates/stretching/yoga Intensity: light to moderate *Sessions with APA teacher*	45 min	45 min	60 min	60 min	60 min	60 min
Cardio fitness Intensity: light *Sessions with APA teacher*			60 min	60 min		
Cardio Intensity: light to moderate *Sessions with APA teacher*					60 min	60 min
Pilates/stretching/yoga + cardio fitness Intensity: light to moderate *Autonomous sessions*				Duration decided by participants		
Total duration of PA from the study	45 min	45 min	120 min	> 120 min	> 120 min	> 120 min

### Initial contact

After the assessment by the APA teacher and before the start of the program, the APA teacher will explain to the participants how the videoconference platform works. He/she will then present the personalized APA program to them.

### Assessment of PA level at inclusion

The PA tests carried out at inclusion will allow the evaluation of the level (beginner, intermediate, expert) of the participants in aerobic activity, mobility, balance, and flexibility.

### APA program

#### Program structure

The 6-month APA program will be supervised via the videoconferencing platform and/or performed independently based on a personalized written program that is updated weekly or bi-weekly by the APA teacher. The program will be based on structured sessions alternating two types of PA: cardio fitness sessions and stretching/yoga/Pilates sessions (to be practiced following the teacher’s oral or written instructions). Each session will last from 45 min to 1 h and will be of low to moderate intensity depending on the level of physical condition at the start. A maximum of 6 participants will be able to attend a session.

The sessions will be structured, adapted, and progressive. During the sessions, the exercises will be adapted according to the means and materials available to the patients at home or in their immediate environment.

#### Structured sessions

Each session will include the following:

1. A warm-up period
2. Stretching, yoga, Pilates, or endurance during the cardio fitness sessions (light to moderate)
3. A recovery period consisting of stretching and relaxation

The duration and intensity of the sessions will be modified according to the physical evaluation and the patients’ declarations at the beginning of each session. In case of intense fatigue and pain, the sessions will be adapted.

The duration and the intensity of the sessions will also evolve according to the capacities of the patients evaluated in a declarative way at the end of each realized session: pain, fatigue, and quality of life.

#### Course of the sessions in autonomy

The patients will be progressively led to practice in an autonomous way (i.e., without supervision) but following an adapted, personalized, and structured program. These PA sessions can be declared afterwards to the PA teacher, specifying the duration of the activity and the exercises actually performed.

### End of the intervention

At the end of the 6-month intervention, participants will keep all records of their written program in order to keep ideas of exercises and to encourage them to continue regular unsupervised exercise on their own. If a contraindication (e.g., injury, not planned but necessary surgery) to PA occurs during the intervention, the participant will be asked to interrupt the program.

### Description of the “education” arm

In addition to the recommendations in terms of PA, participants will benefit from 6 months of education sessions which will include 12 sessions (1-h duration, twice a month) scheduled according to the availability of the participants and by videoconference. Two types of sessions will be proposed: sessions “between participants only” (with the PI), to discuss of general problems linked to endometriosis and tips to manage it, and sessions with an endometriosis specialist (gynecologic surgeon, pain specialist, micronutritionist, physiotherapist). These sessions will help them to better understand endometriosis symptoms and its management. A final individual session will be held at the end of the intervention and will allow for an informal educational assessment.

### Description of the CRESCENDO program: “APA + education” arm

In addition to the PA recommendations, participants in the “APA + education” arm will benefit from both the APA arm and education arm programs.

### Description of the waitlist control group

Participants randomized to this arm will receive written PA recommendations (booklet) and a video of movements to perform, without further intervention. These individuals will receive the PA and education program (3–6 months) at the end of their initial program.

### Criteria for discontinuing or modifying allocated interventions {11b}

If two participants (A and B) live together and are not randomly allocated to the same arm, because of the potential contamination, the second person (B) to be randomized will be allocated to the first participant (A)’s arm. There are no other criteria for discontinuing or modifying allocated interventions. Participants may withdraw voluntary from trial participation at any point. They can give none or any reason to do so. They will be informed that withdrawal from the study has no negative consequences or disadvantages on their care. The PI might also terminate participation if contraindications or severe adverse events arise during the intervention.

### Strategies to improve adherence to interventions {11c}

In addition to the follow-up by the APA teacher for the two arms proposing APA, a telephone or email follow-up will be carried to ensure the good implementation of the program (at 1, 2, 4, and 5 months). Participants will be stimulated and encouraged to remain physically active on a daily basis. Patients will be able to contact PI at any time, by email or by phone. The adherence will be monitored by the attendance at the APA sessions and at the education sessions.

APA teachers will be also given with a notebook about the teaching strategies to motivate participants and maintain their motivation. These strategies are from the self-determination theory [36–38], a theory often used in promotion of PA in several context including health context. This could also limit the “APA teacher” effect which are differences or changes that can be due to the teacher who led the session and not to the intervention.

### Relevant concomitant care permitted or prohibited during the trial {11d}

Any treatment or change in treatment with direct influence on trial outcomes is permitted during the trial, because in endometriosis context, it could take time to find the adequate treatment, and it could be necessary to test and change this treatment every 3 or 4 months.

### Provisions for post-trial care {30}

Participants in the waitlist control group will benefit from APA session after the end of the main study (i.e., 6 months after their inclusion). Participants in APA arm will benefit from the minutes of the education sessions.

## Outcomes {12}

### Primary outcome measure

The aim of the study is will be to investigate potential change in QoL between baseline, middle (3 months) and end of the study (6 months), and 3- and 6-month follow-up. Greater improvements are expected in the APA + education arm compared to the waitlist control group.

#### Quality of life (QoL)

Endometriosis-related QoL will be assessed using the French version of the Endometriosis Health Profile-30 + 23 (EHP-30+23) [39]. The main questionnaire (30 items) is complemented by a 23-item facultative questionnaire with six subscales (treatment, relationship with children, work life, sexual intercourse, medical profession, and infertility). Items are rated on a Likert scale ranging from 0 (never) to 5 (always). A score ranging from 0 to 100 is calculated, with the low the score the better the QoL. The short version (EHP-5) will also be proposed in order to compare its accuracy to the long version.

### Secondary outcome measures

Except for pain, fatigue, and PA which will be assessed at the same points of measurement as the primary outcomes, all secondary outcomes will be assessed at baseline, at the end of the study (6 months), and at 6 months follow-up.

Pain and fatigue will be assessed, separately, using visual analog scales from 0 (no pain or no fatigue) to 10 (pain or fatigue as extreme as possible) [40, 41]. The questions will be about the current pain or fatigue, the pain or fatigue during the last 7 days, and the pain or fatigue during endometriosis crisis. A decrease of 1 point on VAS will be considered as significant [19]^1^.

PA will be assessed using the International Physical Activity Questionnaire (IPAQ, [42]). The IPAQ measures the number of days per week and the time (hours and minutes) spent in vigorous, moderate PA and in walking during past 7 days. The questionnaire also includes questions about time spent in sedentary behaviors. This primary outcome will be defined as the proportion of patients who achieve the internationally recommended level of PA international recommendations, i.e., at least 150 min per week of moderate to vigorous PA [43].

#### Motivational variables

From the theory of planned behaviors (TPB) [44], intention (three items), attitudes (six items), norms (six items), and perceived behavioral control (eight items) will be assessed within 7-point scales [45].

From self-determination theory [36], motivation for PA will be assessed using EMAPS (motivation toward PA un health context scale [Echelle de Motivation envers l’Activité Physique en contexte de Santé] [46]). The 18 items are assessed within a 7-point Likert scale from 1 (does not correspond at all) to 7 (corresponds very strongly). The types of motivation evaluated are intrinsic, integrated, identified, introjected, external regulation, and amotivation.

Basic psychological needs will be also assessed. Twenty-nine items will allow to measure satisfaction, frustration, and unfulfillment of the need for autonomy, competence, and relatedness within a 7-point Likert scale from 1 (strongly disagree) to 7 (strongly agree) [47].

#### Barriers to PA

This questionnaire will evaluate perceived barriers to PA related to health (7 items), beliefs (7 items), environment (10 items), and safety (6 items) within a 5-point Likert scale form 1 (not important) to 5 (important) [48].

#### Kinesiophobia (Tampa)

The fear of being hurt during physical practice will be assessed using 17 items and a 4-point Likert scale from 1 (strongly disagree) to 4 (strongly agree) [49].

#### Stereotype

The questionnaire was adapted from a study on cancer, and PA stereotypes will measure the perception of the ability to endometriosis patients to practice PA within 19 items using a 6-point Likert scale from 1 (not agree at all) to 6 (totally agree) [50].

#### Social support

Social support from friends (12 items), life partner (if relevant, 12 items), and family (15 items) will be assessed within 16 to 39 items using a 5-point Likert scale from 1 (never) to 5 (very often) [51–53].

#### Self-concept

Self-concept about health (8 items), adiposity (6 items), sport skills (6 items), physical appearance (6 items), global physical satisfaction (6 items), and global self-esteem (8 items) will be measured using 5-point Likert scale from 1 (wrong) to 5 (true) [54].

#### Functional capacities

Physical assessment consisted of 2 minutes knee raise test (cardiorespiratory endurance), the Unipedal Stance Test (UPST) (balance and proprioception), Back Scratch Test (high flexibility), 30 s Chair Stand Test (explosive strength of lower limbs), and Sit and Reach (low flexibility).

### Participant timeline {13}

**Fig. 2:**
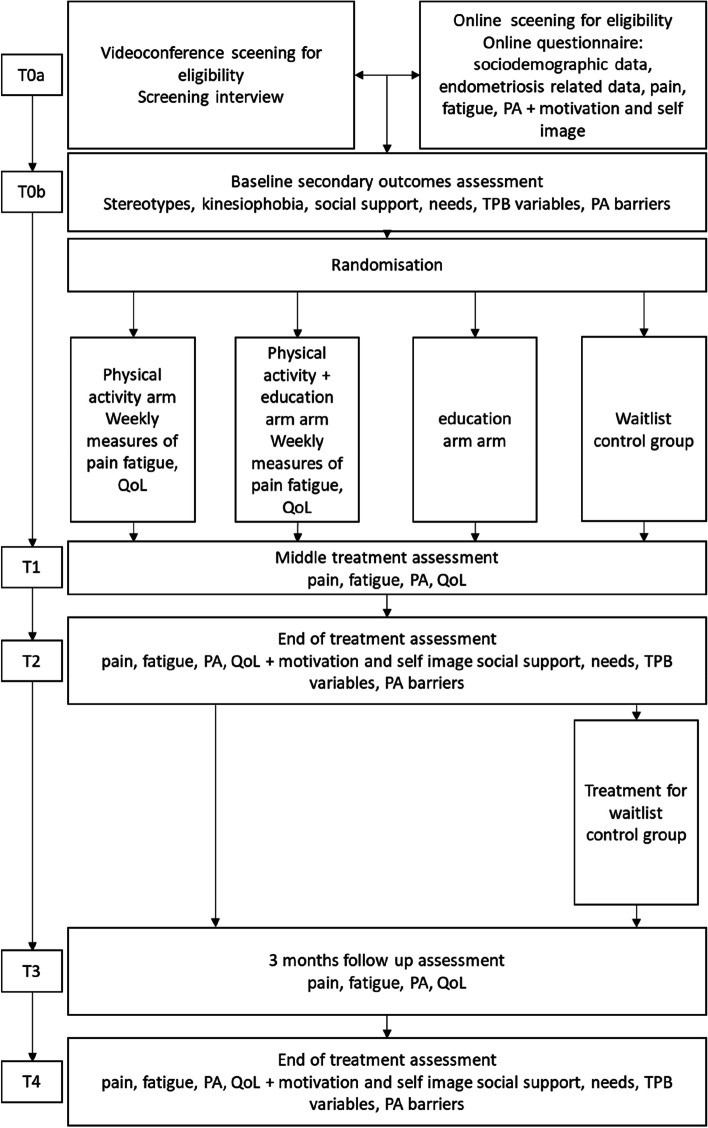
Participant timeline. TPB, theory of planned behaviors; QoL, quality of life

Participant timeline and checkpoints are summarized in Fig. 2.

### Sample size {14}

Two hundred participants will be recruited. This sample size was chosen for several reasons: lack of article on the topic and resource limitations [55]. Except for one, all previous studies recruited or planned to recruit a sample size below 200. Furthermore, the specialists working on the project receive between 5 and 30 endometriosis patients per week. By taking the smallest number of patients and considering the possibility that these patients may refuse to participate or fall into the exclusion criteria, we can assume that only 2 women per week will be included in the study (*N* = 2 × 7 hospitals × 20 weeks of recruitment = 280). Of these participants, 20% may drop out of the study (*N* = 224). We performed sensitivity analyses for repeated measures ANOVA analysis. Effect sizes found were small to medium (*N* = 200 to be more conservative, *α* = 0.05, *β* = 0.90).

### Recruitment {15}

Participants will be recruited online via social media, printed media, radio, and patient associations as well as through the endometriosis specialists who are part of the study. Patients will receive a flyer, and those interested in participating will receive a link, QR code, and the email address of the principal investigator to be able to demonstrate his/her interest.

## Assignment of interventions: allocation

### Sequence generation {16a}

Participants will be randomized to one of the four arms using complete randomization which consist of uniformly distributing them. This procedure is performed using an R script. We opted for this procedure to avoid selection bias as this is the only procedure that does not permit to guess the assignment (Supplementary Material). Moreover, it minimizes various bias and various methods allows to correct the bias provoked by unbalanced in arms [56].

### Concealment mechanism {16b}

Each participant will be entered in the database to be allocated only once the consent form and the baseline online questionnaire are completed and sent to the principal investigator. Thus, each participant will be included chronologically, the date and hour of the last document sent in the principal investigator’s mailbox being the references. The principal investigator is the only one to know when the document was received. Allocation concealment will be ensured as R script will not release the randomization code until the patient has been included into the trial.

### Implementation {16c}

Randomization will be implemented by the principal investigator. Participants will receive an email to inform them about their allocation.

## Assignment of interventions: blinding

### Who will be blinded {17a}

Regarding the design of the intervention, researchers and participants will not be blinded to the allocated trial arm.

### Procedure for unblinding if needed {17b}

As the design is open label, unblinding will not occur.

## Data collection and management

### Plans for assessment and collection of outcomes {18a}

Variables will be assessed (1) online via self-report measures and (2) via the questionnaire proposed by the APA teacher at the beginning and at the end of the APA sessions. Primary outcomes will be assessed at baseline, 3 months, 6 months (end of the study), and at 3 and 6 months’ follow-up for all arms. Secondary outcomes will be assessed at baseline, 6 months (end of the study), and 6 months’ follow-up. Sociodemographic variables will be assessed at baseline only.

### Plans to promote participant retention and complete follow-up {18b}

Participants will be contacted for follow-ups via email. Participants will not be offered additional incentive to complete the trial. However, the questionnaires will be available online, and they are as short as possible which reduces the burden of its completion.

### Data management {19}

The principal investigator will send to each participant a study participant number in order to identify them throughout the trial. The biostatisticians and the principal investigators involved in the study will monitor data entry and check for any missing, abnormal, or extreme data.

### Confidentiality {27}

All data will be stored on local servers of the Upper Alsace University, and the access to the file is restricted to the principal investigator via a key locker according to GDPR policy and the PIA. All data will be kept strictly confidential, and the data from the participant will be pseudonymized (study participant number).

### Plans for collection, laboratory evaluation, and storage of biological specimens for genetic or molecular analysis in this trial/future use {33}

See the “Additional consent provisions for collection and use of participant data and biological specimens {26b}” section; there will be no biological specimens collected.

## Statistical methods

### Statistical methods for primary and secondary outcomes {20a}

General linear models and multilevel analyses comparing the difference in mean scores between groups with repeated measures will be performed. Two-sided *p* < .05 will be considered statistically significant. Moderation and mediation analysis will also be performed. Statistical analysis plan will be adjusted according to the occurrence of unpredictable events. Sensitivity analyses will be performed to investigate the possible influence of missing data on the robustness of the results. Results will be reported according to the CONSORT—Consolidated Standards of Reporting Trials. The statistical analysis plan can be found on osf (https://osf.io/34unj).

### Interim analyses {21b}

No interim analyses are planned.

### Methods for additional analyses (e.g., subgroup analyses) {20b}

If the sample size of 200 participants cannot be reached, the data from APA and APA + education arms will be gathered together, and the same protocol will be planned for control group and education arm. If sociodemographic characteristics differ between arms, subgroup analyses will be performed according to the differences found. However, the sample size and the randomization procedure should prevent these issues.

### Methods in analysis to handle protocol non-adherence and any statistical methods to handle missing data {20c}

Missing data will be explored. Imputation of missing values is not planned in order to analyze “real-life” data.

### Plans to give access to the full protocol, participant-level data, and statistical code {31c}

The present protocol article will be the only one to be published. This article and supplementary material (randomization script) will be made available on an open access platform (e.g., SportRxiv, HAL).

At the end of the study, we will do the same with the article presenting the results, the participants data (which will stay pseudonymized and will not have any sociodemographic information), and the analyses scripts.

## Oversight and monitoring

### Composition of the coordinating center and trial steering committee {5d}

The principal investigator and post-doctoral fellow will be responsible for the design, planning, and conduct of the study, informing the other partners of their missions, preparing the protocol and documents (flyers, questionnaires, consent form, ethics committee application) and their revisions, organizing steering committee meetings, being in charge of the first videoconference to explain the study in detail to each participant and randomization and the collection of signed consent forms, and directing the valorization of results (reports, articles). They will also report any adverse events to the Data Protection Committee.

The principal investigator, the post-doctoral fellow, and two biostatisticians will collect, verify, and analyze the data. In each health center, specialists will provide information about the study to their patients when they meet the inclusion/exclusion criteria. They will also be part of the study’s steering committee, which thus includes gynecological surgeons, endometriosis experts, pain specialists, and APA experts, and will schedule periodic (every 3 months) meetings to first validate the protocol and then review the study’s progress and discuss solutions to overcome any problems or obstacles identified. These people are not part of the sponsor’s services and have no competing interests.

### Composition of the data monitoring committee, its role and reporting structure {21a}

The present study can be acknowledged as a minimal risk study. The data monitoring committee includes two biostatisticians, one endometriosis specialist, and one APA specialist. Even if they will also be part of the steering committee, their role is distant enough from the study to ensure their impartiality. They will meet twice a year, starting with 1 month after the start of the recruitment, and will evaluate the trial data for patient safety, study conduct, and progress and will make recommendations to the steering committee to improve the study. They are not part of the sponsor’s services and have no competing interests.

### Adverse event reporting and harms {22}

Endometriosis specialists and APA teachers will monitor and collect adverse events related or not to the treatment proposed in the study. Also, any detrimental event occurring during the study (e.g., worsening of pain, injury) will be considered as adverse events. These events will be reported to the person protection committee [comité de protection des personnes, CPP].

### Frequency and plans for auditing trial conduct {23}

The trial will be monitored by the principal investigator. The CPP could conduct additional independent auditing.

### Plans for communicating important protocol amendments to relevant parties (e.g., trial participants, ethical committees) {25}

The CPP will be notified for approval of any protocol modifications. Updates of the protocol would be done.

### Dissemination plans {31a}

Trial results will be communicated via articles in scientific journals, on open access and pre-print platforms, on public media, in oral communication during conferences, in patient’s association seminars, and to patients via a report which will be also sent to sponsors.

## Discussion

The aim of the study will be to test the effect of APA and/or endometriosis-based education to reduce pain and fatigue and to increase PA in patients with endometriosis. Given the limited effectiveness of current treatment (pharmaceutical treatment and surgery), in order to find a way to help improving the effectiveness of the treatment particularly on QoL, there is a need for assessing the efficacy of non-medical interventions targeting physical and mental health via a relatively low-cost solution: PA. The objective is not to replace current treatment but to include in the treatment protocol non-intervention which could improve QoL. In order to investigate the effectiveness of the intervention, a waitlist control will be recruited and used as the comparator. It was expected that the combined intervention will have more important effect than other interventions and the control group. The separate interventions (i.e., only APA, only endometriosis-based education) are supposed to be equivalent not only for QoL but also for pain and fatigue, but the APA intervention is supposed to increase PA during and after the intervention, regarding the content of the intervention.

If several strengths of this study could be highlighted, some limitations should be also considered. Investigating four arms this study will inform about the effectiveness of the two-intervention components separately and combined. The sample size will be large enough to obtain reliable results. The videoconference modality will ease the recruitment and the adherence for PA sessions. Study about endometriosis and PA are scarce, and this study will help to partly fill this scientific gap.

The use of video of movement and waiting list could result in a Hawthorne effect. However, to control this effect, the same video will be sent to every participant, and the duration of the study should be sufficient to decrease this potential effect. Otherwise, it seems more ethical to propose to participants in the control group to also benefit from a potentially effective treatment. The videoconference modality and online questionnaire induce a selection bias given that only patients with electronic device, numerical literacy, and Internet access will be able to participate. However, if the program is proven to be effective, it could be easily adapted for an on-site intervention.

## Conclusion

Results from the present study will help to improve care for persons who suffer from endometriosis by informing health professionals about the effectiveness of PA and education to reduce the symptoms and by giving some guidelines and advices to do so.

## Trial status

The trial is registered; trial registration: ClinicalTrials.gov, NCT05831735. Date of registration: April 25, 2023. Recruitment will start in March 2023 and will be completed in December 2024. The current protocol is version 1 (dated April 25, 2023).

### Supplementary Information

**Additional file 1.** Randomization script

## Abbreviations

APA: Adapted physical activity
CPP: [Comité de protection des personnes]
EHP-30 + 23: Endometriosis Health Profile 30 + 23
PA: Physical activity
PI: Principal investigator
QoL: Quality of life

## Footnotes

1 Pain, fatigue, and QoL will also be evaluated at the beginning and at the end of each APA session, each on 10-point VAS
