# Supplementary material for: Effects of a physical activity and endometriosis-based education program delivered by videoconference on endometriosis symptoms: the CRESCENDO program (inCRease physical Exercise and Sport to Combat ENDOmetriosis) protocol study
Source: Trials. 2023 Nov 27;24:759. doi: 10.1186/s13063-023-07792-1 (PMC10680283; doi:10.1186/s13063-023-07792-1)
Supplement: Supplementary file 1 — Additional file 1. Randomization script [file 13063_2023_7792_MOESM1_ESM.docx]

Supplementary material

#nom du fichier avec la liste des particpants Ã  dÃ©clarer ci-dessous

nom_fichier_entree<-"rolling_test.xlsx"

library("readxl")

data<-read_excel(nom_fichier_entree)

#DÃ©cidez d'une graine et ne plus la changer

graine<-7529

set.seed(graine)

for(i in 1:length(data$Nom)){

data[i,3]<-rbinom(1,1,0.5)

data[i,4]<-rbinom(1,1,0.5)

}

#nom du fichier avec la randomisation

nom_fichier_sortie<-"20230303rolling_test_80.xlsx"

library("writexl")

write_xlsx(data,nom_fichier_sortie)
